# The long and winding road: perspectives of people and parents of children with mitochondrial conditions negotiating management after diagnosis

**DOI:** 10.1186/s13023-021-01939-6

**Published:** 2021-07-13

**Authors:** Janet C. Long, Stephanie Best, Sarah Hatem, Tahlia Theodorou, Toni Catton, Sean Murray, Jeffrey Braithwaite, John Christodoulou

**Affiliations:** 1 Australian Institute of Health Innovation, Macquarie University, Sydney, Australia; 2 Australian Genomics Health Alliance, Murdoch Children’s Research Institute, Melbourne, Australia; 3 The Mito Foundation, Sydney, Australia; 4 Department of Paediatrics, Murdoch Children’s Research Institute, University of Melbourne, Melbourne, Australia

**Keywords:** Qualitative research, Rare disease, Management, Consumer experience, Mitochondrial

## Abstract

**Background:**

The diagnostic odyssey for people with a rare disease is well known, but difficulties do not stop at diagnosis. Here we investigate the experience of people, or parents of children with a diagnosed mitochondrial respiratory chain disorder (MRCD) in the management of their disease. The work complements ongoing projects around implementation of consensus recommendations for management of people with MRCD. People with or caring for a child with a formally diagnosed MRCD were invited to take part in an hour-long focus group held via videoconference. Questions elicited experiences of receiving management advice or information specific to their MRCD in four areas drawn from the consensus recommendations: diet and supplements, exercise, access to social services, and mental health. Sessions were audio-recorded, transcribed and analysed using a combination of inductive and deductive coding.

**Results:**

Focus groups were conducted with 20 participants from five Australian states in June–September 2020. Fourteen adults with a MRCD (three of whom also had a child with a MRCD), and six who cared for a child with a MRCD took part. The overarching finding was that of the need for ongoing negotiation to access the advice and service required to manage their condition. The nature of these negotiations varied across contexts but mostly related to joint decision-making, and more commonly, the need to advocate for their care with non-specialist services (e.g., dieticians, schools). The effort required for this self-advocacy was a prominent theme. While most participants reported receiving adequate advice around supplements, and to a lesser extent diet and exercise, the majority reported no formal advice around mental health or practical assistance accessing social services.

**Conclusion:**

These focus groups have revealed several gaps in the system for people with a MRCD, interacting with care providers after diagnosis. Focus group participants had to negotiate with a range of different stakeholders in order to secure appropriate advice or services. Notable was the gap in appropriate generalist services (e.g., dieticians) with sufficient knowledge of MRCD to support people with their day-to-day challenges.

**Supplementary Information:**

The online version contains supplementary material available at 10.1186/s13023-021-01939-6.

## Introduction

The long and winding road that leads to your door

Will never disappear; I’ve seen that road before.

[Lennon and McCartney, Apple Music]

Mitochondrial Respiratory Chain Disorders (MRCDs) are among the most common inborn errors of metabolism, with a conservative estimated incidence for severe MRCD of 1 in 5,000 births [1]. Most of these disorders present during infancy, with a median survival rate of 12 years [2]. Childhood presentations tend to have a more severe and acute phenotype [3], while adult cases often have milder chronic phenotypes [4]. MRCDs can affect any organ, with highly variable clinical presentations, usually involving multiple systems [3, 5, 6].

A major research project is currently underway in Australia to develop a rapid genetic diagnostic process for mitochondrial conditions using next generation sequencing technologies (Australian Genomics Health Alliance Mitochondrial Flagship) [7, 8]. However, an issue that remains is how best to manage patients once a diagnosis has been established.

This challenge is commonly encountered by others with a rare disease. In a systematic review of people’s psychosocial experiences of rare disease [9], 14 of the 21 articles found that meeting health professionals with poor knowledge of their rare diagnosis was common and could result in lack of actionable information, distrust, inappropriate treatments [10, 11], and difficulty in accessing social services [12]. Survey results from 547 people with a rare condition and 214 carers highlighted the importance of support from online and face-to-face peer or charity support groups to alleviate this lack of knowledge [13]. Mental health issues for people with a rare condition is also common, with many patients and carers reporting they had never been asked about their mental health [13, 14].

There are currently very few effective therapies for MRCDs, but there is a level of consensus [15], and some evidence, for treatments that can alleviate symptoms and improve quality of life. Benefits have been associated with interventions around nutrition, supplements and cofactor therapy [16], exercise [17], and active screening for mental health issues [18].

MRCDs are diverse and the initial health issues that patients face are variable, usually differing in paediatric and adult-onset forms of the disorder. Clinical features evolve over time, further complicating approaches to management. It is therefore not surprising that the approaches to management can vary considerably from one clinical centre to another. To address this variability, and the possible sub-optimal care that may result, an international consortium of experts developed recommendations for the management of patients with MRCDs based on best available evidence and consensus of experts [15].

This current study aimed to understand the experience of MRCD management in the Australian context from the perspective of people living with a diagnosed MRCD, or parents of children with a diagnosed MRCD.

## Results

Focus groups were conducted with 20 participants resident in five states of Australia (New South Wales, Victoria, Queensland, South Australia, Western Australia) in June–September 2020. There were 14 participants who had a MRCD and nine participants who cared for a child with a MRCD. Three people with MRCD themselves also cared for a child or children with a MRCD. Two parents had cared for children that had died. Table 1(a) has participant details. Only one enrolled participant did not attend. Surveys were completed by most of the focus group participants; 20 completed the pre-group survey and 13 the post group survey. Table 1(b, c) shows results.

**Table 1:** Details of (a) focus group participants, N = 23, (b) pre-focus group survey responses N = 20 and (c) post-focus group survey responses N = 13

(a) Focus groups	Number of participants (%)
Adults with a MRCD	14/20 (70%)
Parents of a child with a MRCD	9/20 (45%)
Participants with both	3/20 (15%)
Participants from metro regions	15/20 (75%)

(b) Pre-focus group survey	Number of responses, where N = 20 (%)
Length of time engaging with the health system around their/their child’s MRCD	M = 9.8 years (range 1–50 years)
Diagnosed over three years ago	14 (70%)
Participants who see a MRCD service with a multidisciplinary team	9 (45%)

(c) Post-focus group survey	Number of responses, where N = 13 (%)
Participants who answered “Extremely useful” or “Very useful” to *How useful do you think the new guidelines are?*	11/13 (85%)
Participants who responded “yes” to “*Are the guidelines relevant to your situation?*”	11/12 (92%)

### Negotiation of care

The most striking theme to emerge from the focus groups was the ongoing need to negotiate for every aspect of care. In contrast to service provision encounters for people with high prevalence conditions, consultations regarding MRCDs involved a negotiation and sometimes a ‘testing’ between the stakeholders. Negotiation was seen to occur across different settings and between different pairs of the identified stakeholder groups as well: between the patient or parent and the MRCD specialist services, generalist health services, social services, schools, family and friends and informal support services. The nature of these negotiations varied across contexts. For example, with specialist services, negotiations were predominantly around management decisions where there was uncertainty due to a lack of clear evidence. Negotiations with other non-specialist health and social care providers were around advocating for appropriate care. Similar issues arose for both adults and parents. Example quotes are given in Table 2 around these negotiations.

**Table 2:** Example quotes showing negotiation between person/parent of a child with a MRCD and other stakeholder groups

Negotiation of the person/parent of a child with mito and …	Example quote
Mito specialist services	It's been a little back and forth with [mito] specialists trying to get a concrete solution … I'm trying to paste together now a holistic management approach. Yeah. But I find that there’s a lot of back and forth with specialists and all that does is make us walk away more confused actually to be honest. [ParentFG0109P13]
Generalist health services	Facilitator: And is your psychologist part of your mito team or are they somebody that you’ve found yourself? P11: Found myself. Not part of the mito team [at the hospital clinic] but very mito aware. [AdultFG1507P11]
Social services	We had a lot of trouble with that [accessing social support], a lot of trouble. It took us 12 months, firstly we were rejected by the NDIS [National Disability Insurance Scheme]; we appealed, and you know that all takes months and months that was 12 months before we even got any funding to do anything. [ParentFG2306P1]
Work	I have had to work with my manager at work to make sure I’m in a building that has a lift [because] I don’t do stairs, and that I don’t have a significant walk to get to the building. [AdultFG0307P9]
Schools	My kids, they have learning disabilities and the neurologist tried writing to the school to enforce the [teachers] to help monitor the food intake but it's a fight! I'm still actually fighting the school now about food intake and monitoring their health. It's a fight. [ParentFG2306P4]
Family and friends	My wife’s become pretty handy, umm, she now pretty well mows the grass and yesterday she was hanging blinds and all that sort of stuff… I had my father coming down the other week to do palings on the fence, you know, it’s just really fallen back to family. For me personally I’ve had no external avenue to get any assistance. I’ve had to basically just call on family to come and do things. [AdultsFG0307P8]
Informal support services	With rare disease trying to reach out to people the same or similar was very important for us. Trying to find the right things to do and the right support we need. So yeah, absolutely reaching out internationally. [ParentFG2306P2] [We] didn’t get any guidance or anything. Just researched a lot through the Mito Foundation and other support groups that came up about exercise. [ParentFG2306P4]

A range of documents were mentioned by participants as facilitating negotiations. A letter stating the diagnosis was seen as very useful, as were information booklets from the Mito Foundation (e.g., for schools or GPs). Some participants had personal copies of their comprehensive plans of care yet noted that they were rarely looked at by other health providers. Requests, either written or verbal for new health providers to call the MRCD specialist team before initiating any treatment was reported as often ignored.

### Effort of self-advocacy

Considerable effort was reported by participants around negotiating and advocating for their own or their child’s care:

You have to do so much ringing around to find [a physio] that’s even got an inkling of an understanding of what the problem is. [AdultFG0307P8]

I would say it took me a good 12 months to even manage to lodge all the paperwork and report everything required for the [NDIS] plan [for my child with MRCD] and the process itself was incredibly distressing, and I was just lucky that I came across someone who helped me actually navigate that process because it was extremely difficult to lodge the paperwork given how much time we spend in medical and therapy appointments. [ParentFG0109P13]

### Positive interactions

**Fig. 1:**
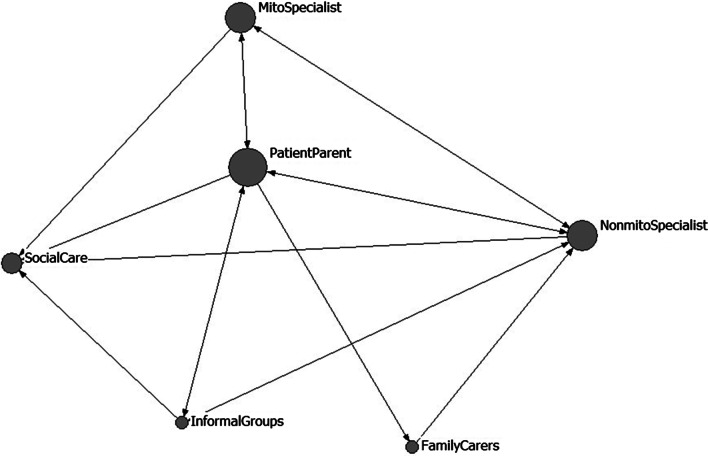
Reported links between different stakeholders in the negotiation of care for a MRCD. Size of the node (each of which represent a different stakeholder group) indicates the frequency of reported links (i.e., larger nodes are involved in more links)

Figure 1 shows a graphical representation of the matrix of all reported positive negotiations regarding management of MRCD. Arrows indicate the direction of the negotiation; i.e., if a patient initiates a negotiation of care with a MRCD specialist, the arrow is shown as going from the patient, pointing to the MRCD specialist. Double ended arrows indicate two-way negotiation. An example of a negotiation between MRCD specialist service and general health provider:

My specialist did write to my personal trainer and said that exercise was important and that I would probably need a sugary snack before I did any exercise. [AdultFG0109P13]

### Missed opportunities to negotiate

**Fig. 2:**
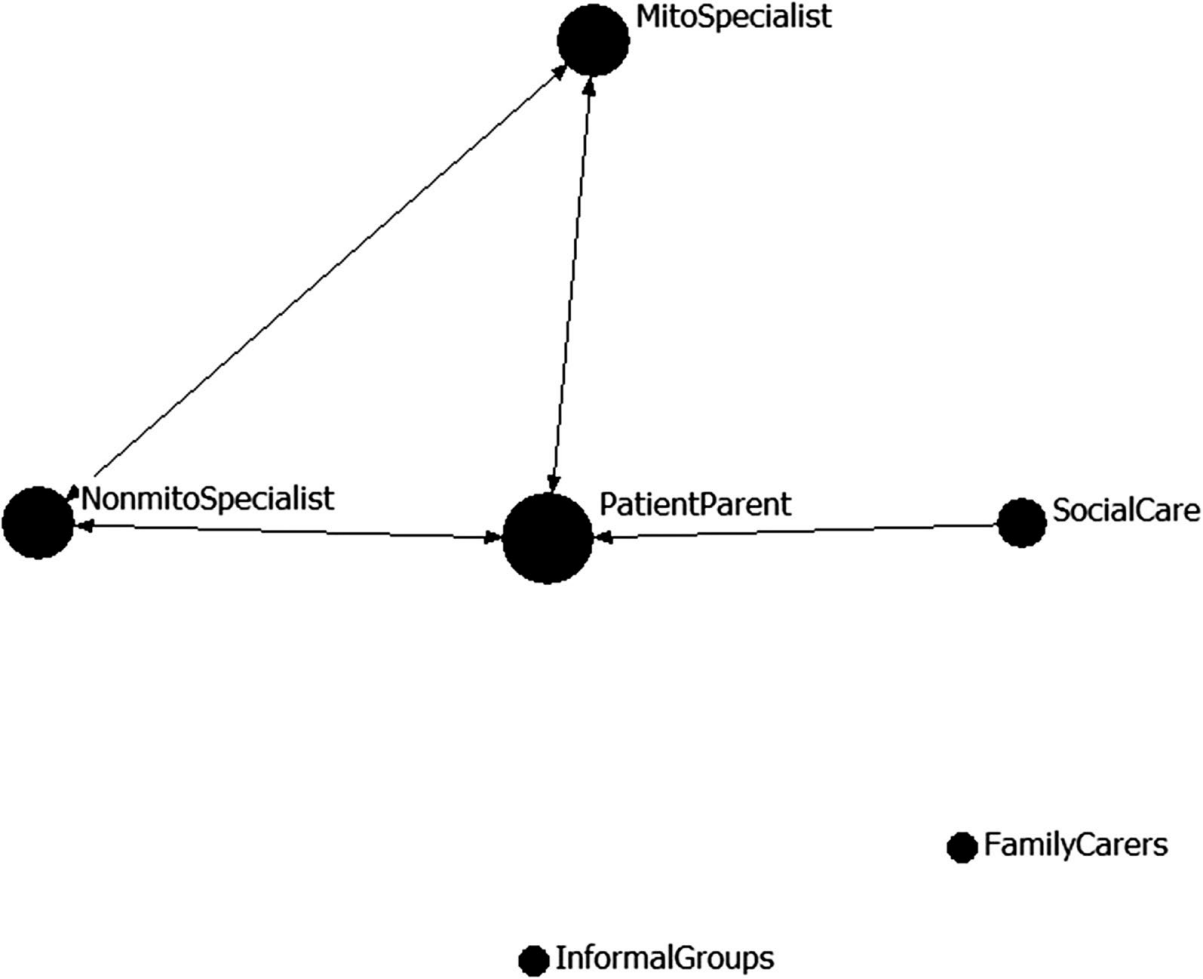
Reported missed links between different stakeholders in the negotiation of care for a MRCD. Size of the node (each of which represent a different stakeholder group) indicates the frequency of the missed opportunities to link (i.e., larger nodes are involved in more missed links). Arrows point to the stakeholder experiencing the negative effect of the missed opportunity

Figure 2 shows a graphical representation of the matrix of all reported negative or missed opportunities to negotiate regarding management of the MRCD. Most missed opportunities were reported between adults or parents of children with MRCD and specialist or generalist health services. However, missed opportunities to access social care had the most negative impact.

I personally drilled my GP to make sure everything we do she uploads onto the master database that doctors and everybody can access. She does her bit, but then I go to the next specialist, or the next mito meeting and they ask me the questions which have already been answered had they taken the time to read this stuff. [AdultFG0307P8]

No, same thing [as the other participants] ... like you ask [about accessing social care] ... but unless you know someone or something then... no... you have to dig really hard to find anything. [AdultFG3006P3]

NDIS only provides… when I said [child 1] had mito, it was “okay well she can have this much” and it was very small. But when I mentioned that [child 2] has autism they said “how much do you want?” I feel like I can manage [the child with autism] but not manage [the child with MRCD]. [ParentsFG0109P14]

We got offered a social worker who added zero value unfortunately, and I just opted out of that. [ParentFG2306P2]

The journey through the NDIS [National Disability Insurance Scheme] was horrific for us. It was terrible. We were never heard. I had to time and time again *prove* why my child needed to be eligible [ParentFG0307P6]

## Cross-cutting themes

A number of themes were identified that were associated in different ways with negotiations with the five stakeholder groups. Table 3 shows the cross-cutting themes by stakeholder negotiation with exemplar quotes to illustrate each.

**Table 3:** Exemplar quotes from the focus groups showing cross-cutting themes within the overarching concept of negotiation of care

	Negotiation with MRCD (“mito”) specialist health services	Negotiation with non-mito specialist health services	Negotiation with social services	Negotiation with informal support networks	Support from family and friends
Impact of cost	The advice I’ve had from the mito professionals has been the CoQ10 [supplement] only. Anything else they have said to me just makes my week really expensive [AdultFG0307P8]	…the cost, and because it seems to be so crucial to the management, it’s just, then this incredible cost that, so I can’t exercise yeah unless it’s supervised, so that’s either with my partner or the exercise physiologist and you know, that’s been difficult and challenging to do [AdultFG0307P9]	… the sort of chronic disease management plans only get you a half a foot through the door really, umm, and there’s after that, unless you’re deemed to have a, meet the criteria for the NDIS, then it’s just out of your pocket. AdultFG0307P9] … overall, the NDIS has been amazing for us because we were struggling to afford the therapies that [my child] needed and so it has been great from that point of view but there are some significant issues with the system. [ParentFG0109P13]		I do think there are benefits to the NDIS, I don’t think that we would be able to manage [financially] as a couple [without it]. I’d feel a bit guilty having to put that on my partner a lot of the time, where already relationships are difficult, so I think like, that would pose a lot more stress on people without NDIS. [AdultFG0307P5]
Uncertainty	You get the feeling that there hasn’t, there’s no sort of consensus on, this is the path we’re going to follow [AdultFG0307P8]	They tried but they didn’t really have much of an idea, though they did try. [AdultFG0307P7]		Ah some of the social forums mention them [supplements]. There’s a lot of mixed messages we’re getting about whether they benefit or they don’t. Some people swear by them, some people can’t see any benefits. [AdultFG0307P5]	Well initially because [child with MRCD] wasn't expected to live there was no plan, and of course when [the child] did live then it was, well, just do the best that you can, so we're talking [many] years ago.[ParentFG2306P3]
Trust	They’ve got a contingency plan of, you know in 12 months from taking that, if that doesn’t work [MRCD specialist] has a plan of [supplement] um, adding that to it [AdultFG0307P9] Participant: Fatigue is an issue which has been much improved by [MRCD specialist’s] suggestion … and that has absolutely revolutionised my life Facilitator: So a lot of your guidance has very much come from [MRCD specialist] and the clinic in [city 1]. So are you staying under their care now you've moved to [city 2]? Participant: Oh yes, I have a telehealth appointment with [MRCD specialist] in a month. [AdultFG0307P12]	[My child] had to go in for tonsils and I said to them “please contact the [MRCD specialist] team regarding anaesthesia and everything else.” “Nah, nah. Don't worry—he'll be fine.” Well, he wasn't fine. [ParentFG2306P4] Everybody is just throwing darts, and oh it might be this, we can try that, the next person it will be something else, and the next person will be something else. [AdultFG0307P8] The hospital physios were amazing and so they were very open to suggestions from the doctors and worked closely with the doctors. It was a very different story when [child with MRCD] hit 2 and we moved outside of the hospital environment to more of those you know therapy focus type organisations [ParentFG0307P5]	The first social worker we had come to the house threw his hands up, had tears in his eyes, and said “I don't even know where to start”, and left because our need by that stage was so great. It was hard and I'm not telling anybody else anything new, it's hard. [ParentFG2306P3]	That's how I found the Mito Foundation—it was through Doctor Google and I'm so grateful I did because they sent out a package, they sent out something for the school, they sent out something for the GP. [ParentFG2306P4]	It’s just really fallen back to family. For me personally I’ve had no external avenue to get any assistance. I’ve had to basically just call on family to come and do things [AdultFG0307P8]
Transition from paed to adult services	The paediatric people are usually the people that know about [the children with MRCD’s] diet, diets and things like that. And that’s the only person we’ve heard of, which is a few hours from us, and yeah, we’ve not been able to get in because [person with MRCD] is 21 [AdultFG0307P7]				
Trial and error	We try and do it all naturally with [certain foods] and stuff like that, we try and hit… and they do a blood test every now and again to see if that's helping the Lactate levels and things like that. So we are measuring as we go along. It's a bit experimental. [ParentFG 0307P5] The advice I was always given was try it [supplement] for six months and then come off and see what happens. [AdultFG0109P13]	I'll never forget the first meeting with a dietitian. She said “if you get this wrong [your child is] going to die”. That was frightening. So yeah I just started asking everyone what do you do? Help! And there was a lot more diagnosed [overseas] and they sent through what they had been given what they've got from their dietitians—what they got, so that's how it all kind of started [ParentsFG2306P4]			My [relative] fortunately, is an ex [nurse] … and she’s taken it upon herself to do a hell of a lot of reading on my behalf and we’ve played a bit of trial and error on a number of different supplements. [AdultFG0307P8]

MRCD = Mitochondrial Respiratory Chain Disorders or “mito disease”; NDIS = National Disability Insurance Scheme)1

### Impact of cost

Many participants talked about out-of-pocket costs arising from positive or negative negotiations with health and social care providers around the management of their MRCD. Costs associated with purchasing recommended supplements, paying for allied health services, and lost earnings were commonly reported.

### Uncertainty

The uncertainty of management options and prognosis was commonly reported. While this understandably often related to generalist health providers who may not have seen a person with a MRCD before, it also included interactions with the more knowledgeable MRCD specialist teams. As well as lack of clinical experience, uncertainty was also linked to a lack of evidence to assist with decision-making.

### Trial and error

Trial and error was commonly reported as an approach to management when evidence was lacking. While some participants accepted this as part and parcel of a rare diagnosis, it caused others considerable distress.

### Trust

There was a mixture of positive and negative sentiments expressed in the theme of trust. Some participants expressed gratitude and confidence in MRCD specialists or generalist health providers with whom they had a good rapport. The Mito Foundation was mentioned in several focus groups as providing trustworthy, high quality information and support. Lack of trust arose from service providers (e.g., specialists not experienced in MRCD) who did not listen to concerns (including failing to accept their diagnosis), or erroneously thought they knew more than they did. Other health providers admitted to being out of their depth and so withdrew their service, breaching the participants’ trust and leaving them without support.

### Transition from paediatric to adult services

As with many other long-term conditions, the transition from paediatric to adult services was generally seen as a negative experience. Parents reported that transitions for their children meant a loss of a ‘holistic’ approach and certain services, only accessible to paediatric patients, were no longer available, e.g., specialist dietician.

### Comments on individual topic areas

Four topic areas (exercise, nutrition and supplements, social care, and mental wellbeing) were used as a framework to guide the discussions of management experiences. Most participants had been advised on the use and (for some) the lack of clear evidence around various supplements. Most participants were familiar with the idea of a “mito cocktail” (a mix of supplements and vitamins with limited high-quality evidence but believed by some to be helpful). Issues around accessing social services were spoken of with the most frustration and passion, with onerous application processes and strong bias of approval for services towards high incidence conditions. Mental health advice had not been given for the majority of participants, often due to it being overshadowed by more pressing physical issues. Three people reported having it raised formally as an issue. Only one of these was given access to a psychologist for their child “from the outset” which they described as “a good experience” [ParentFG2306P1]. Others reported seeking help for themselves or had GPs who facilitated access to counselling.

## Discussion

We held focus groups with twenty people either living with a MRCD, caring for a child with MRCD, or both, exploring their experience of managing their disease after a genetic diagnosis is achieved. The data revealed an overarching concept of an ongoing negotiation of care across contexts: with specialist MRCD services, generalist health services, social service providers, family and friends, and informal support services. Five cross-cutting themes were also found: impact of out-of-pocket costs, trust, uncertainty, trial and error, and transition from paediatric to adult services. Management advice on the four topics was generally good for supplements and diet, slightly less helpful for physical activity, often poor for accessing social care, and virtually non-existent regarding mental health.

The concept of having to negotiate and advocate for one’s care after diagnosis of a rare disease has been reported previously, but studies of this kind are rare. Budych and colleagues noted that there is a well-established information asymmetry between the physician, holding knowledge and skills about the condition, and the patient with a high incidence condition who plays a more passive role [19]. Rare diseases and their accompanying lack of clear evidence around treatment and management options disrupt this dynamic. This can put the patient in the position of having to advocate for their care and become knowledgeable about the condition themselves. The success of this approach is predicated by the physician or other care provider accepting that the patient holds expertise they do not.

Equipping the patient as an expert of their own condition is a key tenet of action and the basis of policies to improve outcomes for people with rare diseases. For example, the European Organisation for Rare Diseases (EURORDIS) states patient empowerment as a key aim: “building the capacities of patients we empower them to become advocates equipped with the knowledge and skills needed to fight for better lives” [20]. Participants in our focus group showed themselves to be very knowledgeable about many aspects of their, or their child’s, condition. Some participants reported respectful and acceptable negotiations using a trial and error approach, in the context of an informed and up-to-date health provider but uncertain evidence. Others reported a failure of health or social care providers to negotiate or even to engage.

Negotiations with the social care provider, the National Disability Insurance Scheme, were mentioned by many participants, mostly expressing frustration. This publicly funded scheme was initiated in 2014 with a staggered implementation across Australia. Its aim is to provide flexible social support packages for people with disabilities [21]. Although considerable efforts considerable efforts have been made by the agency to streamline and simplify the application process, issues persist (e.g., [22]) largely due to algorithms based on “typical” needs of standardised, high incidence conditions [23]. Lack of knowledge about the complex needs of people with a rare disease was reported by our participants as a significant barrier to accessing appropriate levels of social care.

Missed opportunities to interact were reported and were often due to lack of timely or appropriate communication. Lack of communication between different health care providers, even within the same organisation has been reported in other studies (e.g., in the NHS [24]) and is not restricted to communication around people with a rare disease (e.g., people with an acute mental health issue [25]). A patient seeing a health care provider working on their own may be confident of receiving high quality care if their provider follows easily accessible and widely accepted evidence-based guidelines. However, in the context of a rare disease, with little guidance and a management plan that may have to be worked out through trial and error, it is difficult to justify working in isolation. Poorer outcomes can be expected as a result of duplication of assessments or trials and the accompanying waste of time and resources, and decreased patient satisfaction and trust.

In the context of the larger research program that is looking at the active implementation of management guidelines, a clear message from the focus groups was that many of the key management areas affecting the lives of people with a MRCD were not being addressed adequately. Outside of the rare disease context, advice on diet, exercise or mental health is usually referred to appropriate general or allied health practitioners. Here, there was a reported gap and participants spoke about the struggle to find appropriately knowledgeable GPs, exercise physiologists, and dieticians. The gap was often filled by advocacy agency websites such as the Mito Foundation, international Facebook groups and other websites. Advice around accessing social care was mixed. While there were some positive reports of a few MRCD specialist providers who actively facilitated application for NDIS services, a number of participants felt it was left completely to them.

We began with a quote from John Lennon and Paul McCartney’s famous song. That never-ending journey brings to mind both the all-too-common diagnostic odyssey, and also the ongoing journey described here that patients, parents and their children must undergo in the search for continuing care for their condition.

### Limitations

The use of online focus groups may have limited participation by those less digitally confident or equipped. However, it had the advantage of more easily engaging participants from rural and remote areas. There is also the possibility that the topic of the focus groups skewed representation to more proactive and informed people. Recruitment was via the Mito Foundation, so positive comments about that agency’s support should be viewed in that light.

A strength of the study was participants all had a formal diagnosis of MRCD rather than people suspected of MRCD who may still be on the diagnostic odyssey. Questions could therefore concentrate on management across their care journey rather than diagnosis.

## Conclusion

People with a rare disease face many challenges when interacting with care providers after diagnosis. Focus group participants reported having to negotiate with these providers in order to get appropriate advice on management. Success in negotiation was predicated by the health or care provider accepting that there is a different dynamic between them and working alongside them. Most positive interactions were reported with individual MRCD specialist services, individual generalist providers who were seen as going above and beyond, and support groups such as the Mito Foundation. Advice on diet and exercise were most commonly given although follow up with appropriate generalist services (e.g., dietician) was difficult due to lack of knowledge about MRCD disease. Services or providers that facilitated access to social care were greatly appreciated and stood in contrast to the frustration and confusion of those left to work it out themselves. Consistent with other studies in the rare disease field, few participants reported having their mental health formally addressed or assessed at any time.

The strong push from rare disease groups internationally to empower patients to advocate for their own care can only be successful where health providers accept the expertise of the person with a rare disease and agree to work together. Here we see that this is not always the case and the odyssey may continue.

## Methods

This was a mixed methods exploratory study using two short surveys and two parallel sets of focus groups. Ethical approval was given by Royal Children’s Hospital Human Research Ethics Committee (61859/RCHM-2020).

### Focus groups

The consumer advocacy agency, The Mito Foundation facilitated recruitment from across Australia by calling for expressions of interest to take part in a 60-min virtual focus group. Participants were recruited separately for each of the two categories of focus groups: (1) people with a genetically diagnosed MRCD, and (2) parents/carers of children with a genetically diagnosed MRCD. Some participants fit both criteria. We specified “genetically diagnosed” to ensure we recruited people who had interacted with a specialist service and were being managed as a person with a MRCD (rather than suspicion of a MRCD).

A participant information sheet provided general information about the project and the aim. It explained how the group would be run, encouraged respect for the views of the other participants, and stated that comments made in the group would be all de-identified. A brief, plain English summary of the consensus recommendations was provided to give some context, and to frame the questions we asked. We asked participants about advice they had received from health professionals about aspects of their management of their (or their child’s) MRCD, chosen for their applicability to all people with a MRCD. The four areas, taken from the consensus guidelines [15] were: (a) nutrition and supplements, (b) exercise, (c) access to social care, and (d) mental health. We asked who gave them the advice, and the nature of that advice. Participants who fitted the criteria for having a MRCD themselves but also had a child with a MRCD, were asked to consider the questions from the two viewpoints separately. The full schedule of questions is provided in Additional file 1: File #1.

Six to eight participants were recruited into each focus group to give us an optimal number of attendees for the online medium [26]. Participation was voluntary and participants could withdraw at any time without having to give a reason.

As recruitment occurred during the COVID-19 pandemic, all focus groups were held over the video conferencing application, Zoom [27]. Participants joined via a secure link. The focus groups were run by two experienced qualitative researchers (SB, JL) with clinical backgrounds, and the sessions were audio-recorded. The Mito Foundation ensured that a Mito Foundation Helpline resource was available to participants after each focus group for telephone assistance or debriefing if needed.

### Surveys

Participants were emailed an anonymous link to an online survey before the focus group and another after. Each survey took around 10 min to complete and was not a prerequisite for participation in the focus group. The pre-focus group survey collected general demographic data about the participants such as length of time they had been engaging with the health system for their/their child’s MRCD, length of time since diagnosis, which health professional cared for them at the time of diagnosis, and information about their current MRCD specialist care team. The post-focus group survey asked about their impression of the brief summary of the consensus recommendations (see Additional file 2: File #2); specifically, did they make sense, and were they useful?

### Analysis

Data from the focus groups was transcribed verbatim by the research team, imported into QSR International Pty Ltd. Nvivo Qualitatitve Data Analysis and Software Version 12. 2018 for analysis and identifiable features of the experiences, or personal details shared in the group were changed. Transcripts were read for familiarisation and discussed in the coding team (SB, SH, TT and JL) who worked collaboratively over a series of meetings to compile a list of high-level concepts inductively. To aid the analysis, five stakeholder groups who interacted with the focal person with a MRCD were defined: (1) MRCD specialist health providers, (2) non-MRCD specialist health providers (e.g., general practitioners (GPs), physiotherapists, cardiologists), (3) social care providers (e.g., the National Disability Support Service, Home Care), (4) family and friends, and (5) informal support groups (e.g., the Mito Foundation, Facebook groups).

Each of the research team members independently coded a transcript and then discussed findings in the team and considered additional themes. Further discussion led to the identification of an overarching concept of ‘negotiation of care’ enacted within various contexts (e.g., between patient and MRCD specialist team; between MRCD specialist and social care provider), with a set of other themes that were components of these negotiations. Transcripts were then coded deductively (by SH and TT) using this framework. Results were compared and discussed by the coding team as coding proceeded and further insights drawn out. The frequency of negotiations between patients, MRCD services, non-MRCD services, informal support groups, family and friends, and social services were put into a matrix. Frequency of missed opportunities to negotiate were also compiled. These two matrices were converted to graphics using social network software, UCINet [28]. Survey results were analysed using descriptive statistics. Free text answers were used to provide context.

## Supplementary Information

**Additional file 1.** Full schedule of questions used in the focus groups.

**Additional file 2.** Brief summary of the consensus recommendations provided to the focus group participants prior to the session.

**Additional file 3.** Consolidated criteria for reporting qualitative studies (COREQ) checklist.

## Data Availability

Raw data is not available as per Ethical requirements to keep focus group data not identifiable. Focus group schedule, and de-identified quotes are supplied.

## Abbreviations

GP: General practitioner (family physician)
MRCD: Mitochondrial respiratory chain disorder
NDIS: National Disability Support Service

## References

[1] Thorburn DR (2004). Mitochondrial disorders: prevalence, myths and advances. J Inherit Metab Dis.

[2] Darin N, Oldfors A, Moslemi AR, Holme E, Tulinius M (2001). The incidence of mitochondrial encephalomyopathies in childhood: clinical features and morphological, biochemical, and DNA abnormalities. Ann Neurol.

[3] Rahman S, Hanna MG (2009). Diagnosis and therapy in neuromuscular disorders: diagnosis and new treatments in mitochondrial diseases. J Neurol Neurosurg Psychiatry.

[4] Pfeffer G, Chinnery PF (2013). Diagnosis and treatment of mitochondrial myopathies. Ann Med (Helsinki).

[5] Davis RL, Sue CM (2011). The genetics of mitochondrial disease. Semin Neurol.

[6] DiMauro S, Schon EA (2003). Mitochondrial respiratory-chain diseases. N Engl J Med.

[7] Akesson LS, Eggers S, Love CJ, Chong B, Krzesinski EI, Brown NJ (2019). Early diagnosis of Pearson syndrome in neonatal intensive care following rapid mitochondrial genome sequencing in tandem with exome sequencing. Eur J Hum Genet.

[8] Stark Z, Boughtwood T, Phillips P, Christodoulou J, Hansen DP, Braithwaite J (2019). Australian genomics: a federated model for integrating genomics into healthcare. Am J Hum Genetics.

[9] von der Lippe C, Diesen PS, Feragen KB (2017). Living with a rare disorder: A systematic review of the qualitative literature. Mol Genet Genomic Med.

[10] Barlow JH, Stapley J, Ellard DR (2007). Living with haemophilia and von Willebrand's: a descriptive qualitative study. Patient Educ Couns.

[11] Dures E, Morris M, Gleeson K, Rumsey N (2011). The psychosocial impact of Epidermolysis Bullosa. Qual Health Res.

[12] Grut L, Kvam M-H (2013). Facing ignorance: people with rare disorders and their experiences with public health and welfare services. Scand J Disabil Res SJDR.

[13] Rare Diseases UK. Living with a rare condition: the effect on mental health. 2018; Accessed 18 August, 2020. Available from: https://www.raredisease.org.uk/wp-content/uploads/sites/7/2018/07/living-with-a-rare-condition-the-effect-on-mental-health-pdf.pdf

[14] Koerling A-L (2020). No friends 1. Orphanet J Rare Dis.

[15] Parikh S, Goldstein A, Karaa A, Koenig MK, Anselm I, Brunel-Guitton C (2017). Patient care standards for primary mitochondrial disease: a consensus statement from the Mitochondrial Medicine Society. Genet Med.

[16] El-Hattab AW, Hsu JW, Emrick LT, Wong LJC, Craigen WJ, Jahoor F (2012). Restoration of impaired nitric oxide production in MELAS syndrome with citrulline and arginine supplementation. Mol Genet Metab.

[17] Bates MGD, Newman JH, Jakovljevic DG, Hollingsworth KG, Alston CL, Zalewski P (2013). Defining cardiac adaptations and safety of endurance training in patients with m.3243A>G-related mitochondrial disease. Int J Cardiol.

[18] Mancuso M, Mancuso M, Orsucci D, Orsucci D, Ienco EC, Ienco EC (2013). Psychiatric involvement in adult patients with mitochondrial disease. Neurol Sci.

[19] Budych K, Helms TM, Schultz C (2012). How do patients with rare diseases experience the medical encounter? Exploring role behavior and its impact on patient–physician interaction. Health Policy.

[20] Le Cam Y, Bolz-Johnson M, Pomey M-P, Denis J-L, Dumez V (2019). Expert by experience: valuing patient engagement in healthcare. Patient engagement: how patient-provider partnerships transform healthcare organisations.

[21] National Disability Insurance Agency. National Disability Insurance Scheme 2021. Available from: https://www.ndis.gov.au/.

[22] Barr M, Duncan J, Dally K. Parent experience of the national disability insurance scheme (NDIS) for children with hearing loss in Australia. Disabil Soc. 2020:1–25.

[23] King M (2020). Dedifferentiation and difference: people with profound intellectual and multiple disabilities and the National Disability Insurance Scheme (NDIS). J Intellect Dev Disabil.

[24] Crowe AL, McKnight AJ, McAneney H (2019). Communication needs for individuals with rare diseases within and around the healthcare system of northern Ireland. Front Public Health.

[25] Esposito JM, Fein JA, Marshall J, Mitchell C, Aredas B, Zorc JJ. Improving mental health communication from the pediatric emergency department to primary care. 2020;36(9):424–9. 10.1097/PEC.0000000000001928 32870615

[26] Jacqueline MB. Review: Michael Bloor, Jane Frankland, Michelle Thomas, Kate Robson. Focus groups in social research. Forum Qual Soc Res. 2002;3(4).

[27] Zoom Video Communications Inc. Zoom 2016. Available from: https://zoom.us/.

[28] Borgatti SP, Everett MG, Freeman LC. UCInet for windows: software for social network analysis. 6 ed. Harvard: Analytic Technologies; 2002.

