# Supplementary material for: The long and winding road: perspectives of people and parents of children with mitochondrial conditions negotiating management after diagnosis
Source: Orphanet J Rare Dis. 2021 Jul 13;16:310. doi: 10.1186/s13023-021-01939-6 (PMC8276535; doi:10.1186/s13023-021-01939-6)
Supplement: Supplementary file 1 — Additional file 1. Full schedule of questions used in the focus groups. [file 13023_2021_1939_MOESM1_ESM.docx]

Focus Group Schedule

The four areas from the guidelines we would like to discuss are

- Exercise and fatigue
- Supplements and Nutrition
- Accessing Social Care
- Mental wellbeing

| **Area of interest** | **Sample questions** | **Sample prompt** | **Some key findings from the Australian Guidelines** |
| --- | --- | --- | --- |
| **Exercise and fatigue** | Have you been given any advice about exercising and how to manage your fatigue? | - What sort of advice? - From who? - When did you hear this? - Has it been helpful? - What else might be of help? | - There is evidence that aerobic exercise in patients with MDs increases energy production. - Studies have suggested that high-intensity interval training could stimulate mitochondrial biogenesis. However, there is limited evidence for the clinical benefits for this intervention in patients with MDs. |
| **Supplements and Nutrition** | What advice have you been given about nutrition? | - What sort of advice? - From who? - When did you hear this? - Has it been helpful? - What else might be of help? | - There is limited evidence of the benefits of the ketogenic and high fat diets in the context of MDs. - There are anecdotal reports that supplements such as Co Q10, carnitine, thiamine, riboflavin, magnesium orotate, alfa lipoic acid could be helpful. - Supplement carnitine in patients with carnitine deficiency. |
| **Accessing social care** | Have you been given any guidance or support about accessing social care? | - What sort of advice? - From who? - When did you hear this? - Has it been helpful? - What else might be of help? | - Involve a social worker for resource and support planning and to assess for social risk factors that may impact care. A case manager can assist patients in their applications for disability support. |
| **Mental wellbeing** | Have you been assessed for or given any guidance or support about your mental wellbeing? | - What sort of advice? - From who? - When did you hear this? - Has it been helpful? - What else might be of help? | - Routinely screen for depression and anxiety symptoms. - Evaluate psychiatric symptoms promptly as they can be a manifestation of encephalopathy or other change in neurological status. |

**Additional questions**: How do you find the communication between specialist, local consultant and GP? What could improve this?

Who do you go to if you have an acute flare up of your condition? Is this your preferred route? What could improve this?
