# Supplementary material for: The long and winding road: perspectives of people and parents of children with mitochondrial conditions negotiating management after diagnosis
Source: Orphanet J Rare Dis. 2021 Jul 13;16:310. doi: 10.1186/s13023-021-01939-6 (PMC8276535; doi:10.1186/s13023-021-01939-6)
Supplement: Supplementary file 2 — Additional file 2. Brief summary of the consensus recommendations provided to the focus group participants prior to the session. [file 13023_2021_1939_MOESM2_ESM.docx]

# Example recommendations

The consensus-based recommendations are for clinicians who diagnose and manage patients with mitochondrial disease. As mitochondrial disorders can affect any part of the body, in many different ways the guidelines are divided into the sections:

Audiology, cardiology, endocrinology, gastroenterology, haematology, immunology, nephrology, neurology, ophthalmology, orthopaedics, pregnancy, psychiatry, pulmonology. There are also general recommendations for paediatric patients, critical care, altitude, fatigue and exercise, supplements and nutrition and care coordination.

1. **CARDIOLOGY**
   1. Perform baseline cardiology investigations in all patients with suspected or proven Mito Disease (MD). The following baseline assessments should be performed:

- Standard 12-lead electrocardiogram (ECG)
- 24-hour ECG (Holter monitoring) (in adults)
- Echocardiogram (with a measure of left ventricular mass and systolic/diastolic function)
  1. In paediatric patients, perform ECG and echocardiography every 1-2 years to identify subclinical cardiomyopathies.
  2. For patients with Leber’s hereditary optic neuropathy who have been assessed at no risk of cardiac complications, may not require Holter monitoring or echocardiogram.

1. **FATIGUE AND EXERCISE**
2. Evaluate notable or worsening fatigue in MD patients, including for treatable aetiologies: cardiac insufficiency, anaemia, an endocrinopathy (specifically thyroid and adrenal disease), worsening myopathy, respiratory insufficiency, sleep disorder, nutritional and iron deficiencies, and deconditioning.
3. Graded, regular physical activity can improve symptoms of exercise intolerance and fatigability over time.
4. Except for patients with cardiac arrhythmia and/or cardiopulmonary involvement, moderate intensity aerobic exercise at 70% of the patient’s maximum heart rate to improve baseline levels of fitness is recommended.
5. There is evidence that aerobic exercise in patients with MDs increases energy production.
