# Supplementary material for: The long and winding road: perspectives of people and parents of children with mitochondrial conditions negotiating management after diagnosis
Source: Orphanet J Rare Dis. 2021 Jul 13;16:310. doi: 10.1186/s13023-021-01939-6 (PMC8276535; doi:10.1186/s13023-021-01939-6)
Supplement: Supplementary file 3 — Additional file 3. Consolidated criteria for reporting qualitative studies (COREQ) checklist. [file 13023_2021_1939_MOESM3_ESM.docx]

**Supplementary File 3 – Consolidated criteria for reporting qualitative studies (COREQ): 32-item checklist**

Tong A, Sainsbury P, Craig J. Consolidated criteria for reporting qualitative research (COREQ): a 32-item checklist for interviews and focus groups. *International Journal for Quality in Health Care*. 2007. Volume 19, Number 6: pp. 349 – 357

| No. Item | Guide questions/description | Reported on Page # |
| --- | --- | --- |
| Domain 1: Research team and reﬂexivity | | |
| *Personal Characteristics* |  |  |
| 1. Inter viewer/facilitator | Which author/s conducted the interview or focus group? | P.17 |
| 2. Credentials | What were the researcher’s credentials? E.g. PhD, MD | Title page |
| 3. Occupation | What was their occupation at the time of the study? | P.17 |
| 4. Gender | Was the researcher male or female? | NA |
| 5. Experience and training | What experience or training did the researcher have? | P.17 |
| *Relationship with participants* | | |
| 6. Relationship established | Was a relationship established prior to study commencement? | No |
| 7. Participant knowledge of the interviewer | What did the participants know about the researcher? e.g. personal goals, reasons for doing the research | P.16-17 |
| 8. Interviewer characteristics | What characteristics were reported about the interviewer/facilitator? e.g. Bias, assumptions, reasons and interests in the research topic | P.16 |
| Domain 2: study design | | |
| *Theoretical framework* |  |  |
| 9. Methodological orientation and Theory | What methodological orientation was stated to underpin the study? e.g. grounded theory, discourse analysis, ethnography, phenomenology, content analysis | Framework – deductive/inductive analysis |
| *Participant selection* |  |  |
| 10. Sampling | How were participants selected? e.g. purposive, convenience, consecutive, snowball | P.16 |
| 11. Method of approach | How were participants approached? e.g. face-to-face, telephone, mail, email | P.16-17 |
| 12. Sample size | How many participants were in the study? | P.4 |
| 13. Non-participation | How many people refused to participate or dropped out? Reasons? | None – opt in invitation |
| *Setting* |  |  |
| 14. Setting of data collection | Where was the data collected? e.g. home, clinic, workplace | P.17 |
| 15. Presence of non-participants | Was anyone else present besides the participants and researchers? | No |
| 16. Description of sample | What are the important characteristics of the sample? e.g. demographic data, date | P.5 – care was taken to ensure anonymity in this small cohort |
| *Data collection* |  |  |
| 17. Interview guide | Were questions, prompts, guides provided by the authors? Was it pilot tested? | Yes. Additional File 1 |
| 18. Repeat interviews | Were repeat interviews carried out? If yes, how many? | No |
| 19. Audio/visual recording | Did the research use audio or visual recording to collect the data? | Yes P.17 |
| 20. Field notes | Were ﬁeld notes made during and/or after the interview or focus group? | No |
| 21. Duration | What was the duration of the interviews or focus group? | 1 hour |
| 22. Data saturation | Was data saturation discussed? | No |
| 23. Transcripts returned | Were transcripts returned to participants for comment and/or correction? | No |
| Domain 3: analysis and ﬁndings | |  |
| *Data analysis* |  |  |
| 24. Number of data coders | How many data coders coded the data? | P.18 |
| 25. Description of the coding tree | Did authors provide a description of the coding tree? | Yes. P.18 |
| 26. Derivation of themes | Were themes identiﬁed in advance or derived from the data? | Both. P.18 |
| 27. Software | What software, if applicable, was used to manage the data? | NVivo P.17 |
| 28. Participant checking | Did participants provide feedback on the ﬁndings? | No |
| *Reporting* |  |  |
| 29. Quotations presented | Were participant quotations presented to illustrate the themes/ﬁndings? Was each quotation identiﬁed? e.g. participant number | Yes. P.6-11 |
| 30. Data and ﬁndings consistent | Was there consistency between the data presented and the ﬁndings? | Yes. P.6-11 |
| 31. Clarity of major themes | Were major themes clearly presented in the ﬁndings? | Yes. P.6-11 |
| 32. Clarity of minor themes | Is there a description of diverse cases or discussion of minor themes? | Yes. P.6-11 |
